# Dependence of Nanoparticle Toxicity on Their Physical and Chemical Properties

**DOI:** 10.1186/s11671-018-2457-x

**Published:** 2018-02-07

**Authors:** Alyona Sukhanova, Svetlana Bozrova, Pavel Sokolov, Mikhail Berestovoy, Alexander Karaulov, Igor Nabiev

**Affiliations:** 1 Laboratoire de Recherche en Nanosciences, LRN-EA4682, Université de Reims Champagne-Ardenne, 51100 Reims, France; 2 Laboratory of Nano-Bioengineering, National Research Nuclear University MEPhI (Moscow Engineering Physics Institute), 31 Kashirskoe shosse, Moscow, Russian Federation 115521; 3 Department of Clinical Immunology and Allergology, I.M. Sechenov First Moscow State Medical University, Moscow, Russian Federation 119992

**Keywords:** Nanoparticles, Quantum dots, Nanotoxicity, Surface chemistry, Theranostics, Imaging

## Abstract

Studies on the methods of nanoparticle (NP) synthesis, analysis of their characteristics, and exploration of new fields of their applications are at the forefront of modern nanotechnology. The possibility of engineering water-soluble NPs has paved the way to their use in various basic and applied biomedical researches. At present, NPs are used in diagnosis for imaging of numerous molecular markers of genetic and autoimmune diseases, malignant tumors, and many other disorders. NPs are also used for targeted delivery of drugs to tissues and organs, with controllable parameters of drug release and accumulation. In addition, there are examples of the use of NPs as active components, e.g., photosensitizers in photodynamic therapy and in hyperthermic tumor destruction through NP incorporation and heating. However, a high toxicity of NPs for living organisms is a strong limiting factor that hinders their use in vivo. Current studies on toxic effects of NPs aimed at identifying the targets and mechanisms of their harmful effects are carried out in cell culture models; studies on the patterns of NP transport, accumulation, degradation, and elimination, in animal models. This review systematizes and summarizes available data on how the mechanisms of NP toxicity for living systems are related to their physical and chemical properties.

## Background

The International Organization for Standardization define nanoparticles (NPs) as structures whose sizes in one, two, or three dimensions are within the range from 1 to 100 nm. Apart from size, NPs may be classified in terms of their physical parameters, e.g., electrical charge; chemical characteristics, such as the composition of the NP core or shell; shape (tubes, films, rods, etc.); and origin: natural NPs (NPs contained in volcanic dust, viral particles, etc.) and artificial NPs, which are the focus of this review.

Nanoparticles have become widely used in electronics, agriculture, textile production, medicine, and many other industries and sciences. NP toxicity for living organisms, however, is the main factor limiting their use in treatment and diagnosis of diseases. At present, researchers often face the problem of balance between the positive therapeutic effect of NPs and side effects related to their toxicity. In this respect, the choice of an adequate experimental model for estimating toxicity between in vitro (cell lines) and in vivo (experimental animals) ones is of paramount importance. The NP toxic effects on individual cell components and individual tissues are easier to analyze in in vitro models, whereas in vivo experiments make it possible to estimate the NP toxicity for individual organs or the body as a whole. In addition, the possible toxic effect of NPs depends on their concentration, duration of their interaction with living matter, their stability in biological fluids, and the capacity for accumulation in tissues and organs. Development of safe, biocompatible NPs that can be used for diagnosis and treatment of human diseases can only be based on complete understanding of the interactions between all factors and mechanisms underlying NP toxicity.

### Medical Applications of Nanoparticles

In medicine, NPs can be used for diagnostic or therapeutic purposes. In diagnosis, they can serve as fluorescent labels for detection of biomolecules and pathogens and as contrast agents in magnetic resonance and other studies. In addition, NPs can be used for targeted delivery of drugs, including protein and polynucleotide substances; in photodynamic therapy and thermal destruction of tumors, and in prosthetic repair [1–6]. Some types of NPs are already successfully used in clinic for drug delivery and tumor cell imaging [7–9].

Examples of the use of gold NPs have been accumulating recently. They have proved to be efficient carriers of chemotherapeutics and other drugs. Gold NPs are highly biocompatible; however, although gold as a substance is inert towards biological objects, it cannot be argued that the same is true for gold NPs, since there are no conclusive data yet on the absence of delayed toxic effects [10]. In addition to gold NPs, those based on micelles, liposomes [11], and polymers with attached “capture molecules” [12] are already used as drug carriers. Single- and multiwalled nanotubes are good examples of NPs used for drug delivery. They are suitable for attaching various functional groups and molecules for targeted delivery, and their unique shape allows them to selectively penetrate through biological barriers [13]. The use of NPs as vehicles for drugs enhances the specificity of delivery and decreases the minimum amount of NPs necessary for attaining and maintaining the therapeutic effect, thereby reducing the eventual toxicity. This is especially important in the case of highly toxic and short-lived chemo- and radiotherapeutic agents [14].

Quantum dots (QDs) constitute another group of NPs with a high potential for clinical use. QDs are semiconductor nanocrystals from 2 to 10 nm in size. Their capacity for fluorescence in different spectral regions, including the infrared one [15], makes them suitable for labeling and imaging cells, cell structures, or pathogenic biological agents, as well as various processes in cells, tissues, and body as a whole [16–18], which has important diagnostic implications [19, 20]. NPs based on superparamagnetic iron oxide are efficiently used as contrast agents in magnetic resonance tomography (MRT) for imaging liver, bone marrow, and lymph node tissues [21]. There is also an example where radioactively labeled single-walled carbon nanotubes functionalized with phospholipids were used for labeling integrin-containing tumors and their subsequent detection by means of positron emission tomography in experiments on mice [22].

Nanoparticles have also been used in designing biosensors, including those based on carbon nanotubes for measuring the glucose level [23], detecting specific DNA fragments and regions [24], and identifying bacterial cells [25].

Silver (or silver-containing) NPs exert antimicrobial and cytostatic effects; for this reason, they are widely used in medicine, e.g., for treating bandages, surgical instruments, prostheses, and contraceptives [13, 22]. Silver NPs have been reported to serve as effective and safe preservation agents in the cosmetic industry [26].

However, NPs may still be highly toxic, even if the safety of using many of their chemical constituents in medicine has been proved. The toxic effect may be caused by their unique physical and chemical properties, which underlie specific mechanisms of interaction with living systems. In general, this determines the importance of studying the causes and mechanisms of the potential toxic effect of NPs.

### Mechanisms of Nanoparticle Toxicity

The toxicity of NPs is largely determined by their physical and chemical characteristics, such as their size, shape, specific surface area, surface charge, catalytic activity, and the presence or absence of a shell and active groups on the surface.

The small size of NPs allows them to penetrate through epithelial and endothelial barriers into the lymph and blood to be carried by the bloodstream and lymph stream to different organs and tissues, including the brain, heart, liver, kidneys, spleen, bone marrow, and nervous system [27, 28], and either be transported into cells by transcytosis mechanisms or simply diffuse into them through the cell membrane. Nanomaterials can also increase access to the blood stream through ingestion [29, 30]. Some nanomaterials can penetrate the skin [31, 32] and even greater microparticles can penetrate skin when it is flexed [33]. Nanoparticles, because of their small size, can extravasate through the endothelium in inflammatory sites, epithelium (e.g., intestinal tract and liver), tumors or penetrate microcapillaries [34]. Experiments modeling the toxic effects of NPs on the body have shown that NPs cause thrombosis by enhancing platelet aggregation [35], inflammation of the upper and lower respiratory tracts, neurodegenerative disorders, stroke, myocardial infarction, and other disorders [36–38]. Note that NPs may enter not only organs, tissues, and cells, but also cell organelles, e.g., mitochondria and nuclei; this may drastically alter cell metabolism and cause DNA lesions, mutations, and cell death [39].

The toxicity of QDs has been shown to be directly related to the leakage of free ions of metals contained in their cores, such as cadmium, lead, and arsenic, upon oxidation by environmental agents. QDs may be absorbed by mitochondria and cause morphological changes and dysfunction of the organelles [40]. Entry of cadmium-based QDs into cells and formation of free Cd^2+^ ions causes oxidative stress [41, 42].

Recent studies have shown that contact of lung tissue with NPs about 50 nm in size leads to perforation of the membranes of type I alveolar cells and the resultant entry of the NPs into the cells. This, in turn, causes cell necrosis, as evidenced by the release of lactate dehydrogenase [43]. There is evidence that QD penetration increases the cell membrane fluidity [44]. On the other hand, the formation of reactive oxygen species (ROS) induced by peroxidation of membrane lipids may lead to the loss of membrane flexibility, which, as well as an abnormally high fluidity, inevitably results in cell death.

Interaction of NPs with the cytoskeleton may also damage it. For example, TiO_2_ NPs induce conformational changes in tubulin and inhibit its polymerization [45], which disturbs intracellular transport, cell division, and cell migration. In human umbilical vein endothelial cells (HUVECs), damage of the cytoskeleton hinders the maturation of coordination adhesive complexes which link the cytoskeleton to the extracellular matrix, thereby disturbing the formation of the vascular network [46].

In addition, the NP cytotoxicity may interfere with cell differentiation and protein synthesis, as well as activate proinflammatory genes and synthesis of inflammatory mediators. It should be specially noted that normal protective mechanisms do not affect NPs; macrophage uptake of large PEGylated nanoparticles is more efficient than uptake of small ones, which leads to accumulation of NPs in the body [47]. Superparamagnetic iron oxide NPs have been demonstrated to disturb or entirely suppress osteogenic differentiation of stem cells and activate the synthesis of signal molecules, tumor antigens, etc. [48, 49]. In addition, interaction of NPs with the cell enhances the expression of the genes responsible for the formation of lysosomes [50], disturbs their functioning [51], and inhibits protein synthesis [52, 53]. A study on the toxic effects of NPs of different compositions on lung epithelial cells and human tumor cell lines has shown that NPs stimulate the synthesis of inflammation mediators, e.g., interleukin 8 [54]. According to Park, who studied the expression of proinflammatory cytokines in vitro and in vivo, the expressions of interleukin 1 beta (IL-1β) and tumor necrosis factor alpha (TNFα) are enhanced in response to silicon NPs [55].

Oxidation, as well as action of various enzymes on the shell and surface of NPs, results in their degradation and release of free radicals. In addition to the toxic effect of free radicals expressed as oxidation and inactivation of enzymes, mutagenesis, and disturbance of chemical reactions leading to cell death, degradation of NPs leads to alteration or loss of their own functionality (e.g., the loss of the magnetic moment and the changes in the fluorescence spectrum and transport or other functions) [56, 57].

**Fig. 1:**
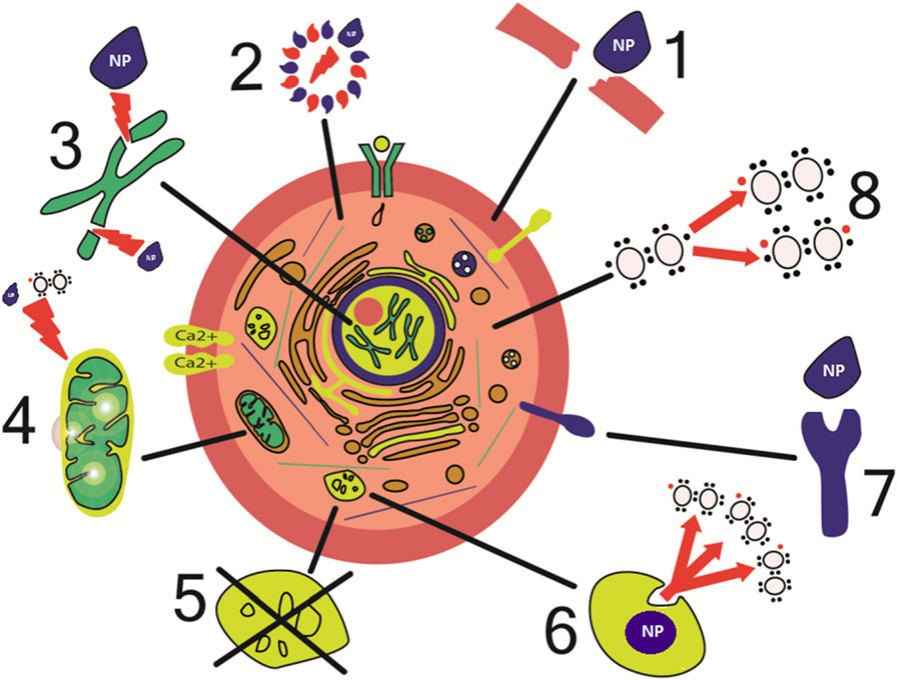
Mechanisms of cell damage by nanoparticles. (1) Physical damage of membranes [43, 67, 75]. (2) Structural changes in cytoskeleton components [45, 46]. (3) Disturbance of transcription and oxidative damage of DNA [61, 62]. (4) Damage of mitochondria [39, 40]. (5) Disturbance of lysosome functioning [51]. (6) Generation of reactive oxygen species [61]. (7) Disturbance of membrane protein functions [172]. (8) Synthesis of inflammatory factors and mediators [54, 55]

In summary, the most common mechanisms of NP cytotoxicity are the following:

1. NPs may cause oxidation via formation of ROS and other free radicals;
2. NPs may damage cell membranes by perforating them;
3. NPs damage components of the cytoskeleton, disturbing intracellular transport and cell division;
4. NPs disturb transcription and damage DNA, thus accelerating mutagenesis;
5. NPs damage mitochondria and disturb their metabolism, which leads to cell energy imbalance;
6. NPs interfere with the formation of lysosomes, thereby hampering autophagy and degradation of macromolecules and triggering the apoptosis;
7. NPs cause structural changes in membrane proteins and disturb the transport of substances into and out of cells, including intercellular transport;
8. NPs activate the synthesis of inflammatory mediators by disturbing the normal mechanisms of cell metabolism, as well as tissue and organ metabolism (Fig. 1).

Although there are numerous mechanisms of NP toxicity, it is necessary to determine and classify the type and mechanism of each particular toxic effect of NPs as dependent on their physical and chemical properties.

### Relationships of Nanoparticle Toxicity with Their Physical and Chemical Properties

The toxicity of NPs is considered to depend on their physical and chemical characteristics, including the size, shape, surface charge, chemical compositions of the core and shell, and stability. In particular, Oh et al., using the data meta-analysis of 307 papers describing 1741 cell viability-related data samples, recently analyzed the CdSe quantum dot toxicity. It has been shown that the QD nanotoxicity is closely correlated with their surface properties (including shell, ligand, and surface modifications), diameter, toxicity assay type used, and the exposure time [58]. Which of these factors is the most important is determined by the specific experimental task and model; therefore, we will now consider each factor separately.

#### Nanoparticle Size and Toxicity

The NP size and surface area play an important role, largely determining the unique mechanism of NP interaction with living systems. NPs are characterized by a very large specific surface area, which determines their high reaction capacity and catalytic activity. The sizes of NPs (from 1 to 100 nm) are comparable with the size of protein globules (2–10 nm), diameter of DNA helix (2 nm), and thickness of cell membranes (10 nm), which allows them to easily enter cells and cell organelles. For example, Huo et al. have demonstrated that gold NPs no larger than 6 nm effectively enter the cell nucleus, whereas large NPs (10 or 16 nm) only penetrate through the cell membrane and are found only in the cytoplasm. This means that NPs several nanometers in size are more toxic than 10 nm or larger NPs, which cannot enter the nucleus [59]. Pan et al. have traced the dependence of the toxicity of gold NPs on their size in the range from 0.8 to 15 nm. The NPs 15 nm in size have been found to be 60 times less toxic than 1.4-nm NPs for fibroblasts, epithelial cells, macrophages, and melanoma cells. It is also noteworthy that 1.4-nm NPs cause cell necrosis (within 12 h after their addition to the cell culture medium), whereas 1.2-nm NPs predominantly cause apoptosis [60]. These data suggest not only that NPs can enter the nucleus, but also that the correspondence of the geometric size of NPs (1.4 nm) to that of the major groove of DNA allows them to effectively interact with the negatively charged sugar–phosphate DNA backbone and block the transcription [61, 62].

In addition, the NP size largely determines how the NPs interact with the transport and defense systems of cells and the body. This interaction, in turn, affects the kinetics of their distribution and accumulation in the body. The review paper by [63] presents both theoretical considerations and numerous experimental data demonstrating that NPs smaller than 5 nm usually overcome cell barriers nonspecifically, e.g., via translocation, whereas larger particles enter the cells by phagocytosis, macropinocytosis, and specific and nonspecific transport mechanisms. An NP size of about 25 nm is believed to be optimal for pinocytosis, although this also strongly depends on the cell size and type [63, 64]. In vivo experiments have shown that NPs smaller than 10 nm are rapidly distributed among all organs and tissues upon intravenous administration, whereas most larger NPs (50–250 nm) are found in the liver, spleen, and blood [65]. This suggests that large NPs are recognized by specific defense systems of the body and absorbed by the system of mononuclear phagocytes, which prevents them from entering other tissues. In addition, Talamini et al. claimed that the NP size and shape influence the kinetics of accumulation and excretion of gold NPs in filter organs, and only star-like gold NPs are able to accumulate in the lung. They have also shown that the changes in the NP geometry do not improve the NP passage of the blood–brain barrier [66].

The large specific surface area ensures effective adsorption of NPs on the cell surface. This was shown in a study on the hemolytic activity of 100- to 600-nm mesoporous silicon particles towards human erythrocytes [67]. The particles 100 nm in size were effectively adsorbed on the erythrocyte surface without causing cell destruction or any morphological changes in the cells, whereas 600-nm particles deformed the membrane and entered the cells, resulting in erythrocyte destruction (hemolysis) [67].

#### Nanoparticle Shape and Toxicity

The characteristic shapes of NPs are spheres, ellipsoids, cylinders, sheets, cubes, and rods. NP toxicity strongly depends on their shape. This has been shown for numerous NPs of different shapes and chemical compositions [68–71]. For example, spherical NPs are more prone to endocytosis than nanotubes and nanofibers [72]. Single-walled carbon nanotubes have been found to more effectively block calcium channels compared to spherical fullerenes [73].

Comparison of the effects of hydroxyapatite NPs with different shapes (needle-like, plate-like, rod-like, and spherical) on cultured BEAS-2B cells have shown that plate-like and needle-like NPs cause death of a larger proportion of cells than spherical and rod-like NPs [74]. This is partly accounted for by the capacity of plate-like and needle-like NPs for damaging cells and tissue upon direct contact. Hu et al. [75] obtained interesting data when studying the damage of mammalian cells by graphene oxide nanosheets. The toxicity of these NPs was determined by their shape allowing them to physically damage the cell membrane. However, their toxicity was found to decrease with an increase in the fetal calf serum concentration in the culture medium. This was explained by a high capacity of graphene oxide NPs for adsorbing protein molecules, which cover the NP surface, thereby changing the shape of the NPs and partly preventing the damage of cell membranes [75].

#### Nanoparticle Chemical Composition and Toxicity

Although the toxicity of NPs strongly depends on their size and shape, the influence of other factors, such as the NP chemical composition and crystal structure, should not be disregarded. Comparison of the effects of 20-nm silicon dioxide (SiO_2_) and zinc oxide (ZnO) NPs on mouse fibroblasts has shown that they differ in the mechanisms of toxicity. ZnO NPs cause oxidative stress, whereas SiO_2_ NPs alter the DNA structure [76].

The toxicity of NPs is indeed largely determined by their chemical composition. It has been shown that degradation of NPs can occur, and its extent depends on the environment conditions, e.g., pH or ionic strength. The most common cause of the toxic effect of NPs interacting with cells is leakage of metal ions from the NP core. The toxicity also depends on the composition of the core of NPs. Some metal ions, such as Ag and Cd, are in fact toxic and, therefore, cause damage of the cells. Other metal ions, such as Fe and Zn, are biologically useful, but, at high concentrations, they could damage cellular pathways and, hence, cause high toxicity. However, this effect can be decreased, e.g., by coating NP cores with thick polymer shells, silica layers, or gold shells instead of short ligands or by using nontoxic compounds for NP synthesis. On the other hand, the composition of the core could be altered by addition of other metals. This can result in enhanced chemical stability against NP degradation and metal ion leakage into the body [77].

The toxicity of NPs also depends on their crystal structure. The relationship between crystal structure and toxicity has been studied using a human bronchial epithelium cell line and titanium oxide NPs with different types of crystal lattice. It has been demonstrated that NPs with a rutile-like crystal structure (prism-shaped TiO_2_ crystals) cause oxidative damage of DNA, lipid peroxidation, and formation of micronuclei, which indicates abnormal chromosome segregation during mitosis, whereas NPs with anatase-like crystal structure (octahedral TiO_2_ crystals) of the same size are nontoxic [78]. It should be noted that the NP crystal structure may vary depending on the environment, e.g., upon interaction with water, biological fluids, or other dispersion media. There is evidence that the crystal lattice of ZnS NPs is rearranged into a more ordered structure upon contact with water [79].

#### Nanoparticle Surface Charge and Toxicity

The surface charge of NPs plays an important role in their toxicity, because it largely determines the interactions of NPs with biological systems [80, 81].

NP surfaces and their charges could be modified by grafting differently charged polymers. PEG (polyethylene glycol) or folic acid is often used to improve the NP intracellular uptake and ability to target specific cells [82]. The synthesis of biocompatible TiO2 nanoparticles containing functional NH2 or SH groups has also been reported [83]. Other substances, such as methotrexate, polyethyleneimine, and dextran, had also been used to modify NP surfaces and their charge [84].

A high toxicity of positively charged NPs is explained by their ability to easily enter cells, in contrast to negatively charged and neutral NPs. This is accounted for by electrostatic attraction between the negatively charged cell membrane glycoproteins and positively charged NPs. Comparison of the cytotoxic effects of negatively and positively charged polystyrene NPs on HeLa and NIH/3T3 cells has shown that the latter NPs are more toxic. This is not only because positively charged NPs more effectively penetrate through the membrane, but also because they are more strongly bound to the negatively charged DNA, causing its damage and, as a result, prolongation of the G0/G1 phase of the cell cycle. Negatively charged NPs have no effect on the cell cycle [85]. Similar results have been obtained for positively and negatively charged gold NPs, positive NPs being absorbed by cells in larger amounts and more rapidly than negative ones and being more toxic [86].

Positively charged NPs have an enhanced capacity for opsonization, i.e., adsorption of proteins facilitating phagocytosis, including antibodies and complement components, from blood and biological fluids [87]. The adsorbed proteins, referred to as the protein crown, may affect the surface properties of NPs. For example, they may alter the surface charge, aggregation characteristics, and/or hydrodynamic diameter of NPs. In addition, adsorption of proteins on the NP surface leads to their conformational changes, which may decrease or completely inhibit the functional activities of the adsorbed proteins. The protein crown mainly consists of major serum proteins, such as albumin, fibrinogen, and immunoglobulin G, as well as other effector, signal, and functional molecules [88, 89]. Binding to NPs alters the protein structure, which leads to the loss of their enzymatic activity, disturbance of biological processes, and precipitation of ordered polymeric structures, e.g., amyloid fibrils [90]. This may lead to various diseases, such as amyloidosis. In vitro experiments have demonstrated that QDs coated with a hydrophilic polymer accelerate the formation of fibrils of human β_2_ microglobulin, which are then arranged into multilayered structures on the particle surface; this results in a local increase in the protein concentration on the NP surface, precipitation, and formation of oligomers [91].

Xu et al. developed a method for changing the NP charge from negative to positive via various modifications of the surface. For example, polymer NPs were modified with a pH-sensitive polymer so that, being negatively charged in a neutral medium, they acquired a positive charge in an acid medium, at pH 5–6 [92]. This technique makes it possible to substantially increase the rate of NP uptake by cells, which could be used for drug delivery to tumor cells. Estimation of the cytotoxicity of surface-modified cerium oxide NPs for H9C2, HEK293, A549, and MCF-7 cells has shown that basically different biological and toxic effects can be obtained by using different polymers to make the NPs positively or negatively charged or neutral. Specifically, positively charged and neutral NPs are absorbed by all cell types at the same rate, whereas negatively charged ones predominantly accumulate in tumor cells [93]. Thus, modification of the NP charge allows their localization and toxicity to be controlled, which could be used for developing effective systems for delivery of chemotherapeutic drugs to tumors.

#### Nanoparticle Shell and Toxicity

Application of a shell onto the surface of NPs is necessary for changing their optical, magnetic, and electrical properties; it is used for improving NP biocompatibility and solubility in water and biological fluids by decreasing their aggregation capacity, increasing their stability, etc. Thus, the shell decreases the toxicity of NPs and provides them with the capacity for selective interaction with different types of cells and biological molecules. In addition, the shell considerably influences the NP pharmacokinetics, changing the patterns of NP distribution and accumulation in the body [94].

As noted above, NP toxicity is largely related to the formation of free radicals [40, 57, 95, 96]. However, the shell can considerably mitigate or eliminate this negative effect, as well as stabilize NPs, increase their resistance to environmental factors, decrease the release of toxic substances from them, or make them tissue-specific [97]. For example, Cho et al. modified polymer NPs by coating them with lectins. The modified NPs selectively bound with tumor cells presenting sialic acid molecules on the surface, which made the NPs suitable for specifically labeling cancer cells [98].

The NP surface can be modified with both organic and inorganic compounds, e.g., polyethylene glycol, polyglycolic acid, polylactic acid, lipids, proteins, low molecular weight compounds, and silicon. This variety of modifiers makes it possible to form complex systems on the NP surface for changing the NP properties and for their specific transport and accumulation.

Nanoparticles coated with shells of synthetic polymers are used for delivery of antigens, thus serving as adjuvants boosting the immune response. This allows obtaining vaccines against the antigens that are targets of strong natural nonspecific cellular immunity [99].

The shell is often used for improving solubilization and decreasing the toxicity of QDs, because their metal cores are hydrophobic and mainly consist of toxic heavy metals, such as cadmium, tellurium, and mercury. The shell increases the stability of the QD core and prevents its desalination and oxidative or photolytic degradation. This, in turn, decreases the leakage of metal ions outside of the QD core and, hence, the toxicity of QDs [100–102].

### Study of Nanoparticle Toxicity

During the past two decades, the use of NPs has tremendously extended and led to the foundation of nanotoxicology, a new science studying the potential toxic effects of NPs on biological and ecological systems. The general goal of nanotoxicology is to develop the rules of synthesis of safe NPs [103]. This calls for a comprehensive, systemic approach to analysis of the toxic properties of NPs and their effects on cells, tissues, organs, and the body as a whole.

There are two routine approaches to the study of the effects of various substances on living systems, which are also applicable to NP toxic effects: in vitro experiments on model cell lines and in vivo experiments on laboratory animals. We do not consider here the third possible approach to estimating NP toxicity, computer simulation, because the pathways and mechanisms of the toxic effects of NPs are not known well enough for a computer model to predict the consequences of interactions between NPs and living matter for a wide range of NPs with sufficient reliability.

Both cell culture and animal experimental models for studying NP toxicity have their specific advantages and disadvantages. The former allow deeper insight into the molecular mechanisms of toxicity and identification of the primary targets of NPs; however, the patterns of the distribution of NPs in the body and their transport to different tissues and cells are not taken into consideration. The study of NP toxicity in animal experiments allows the delayed effects of NP action in vivo to be estimated. However, the general pattern of toxicity manifestations becomes so complicated that it is impossible to determine which of them is the primary cause of the observed effect and which are its consequences.

#### Study of Toxicity in Cell Cultures

Many studies of NP toxicity are carried out in primary cell cultures serving as models of various types of human and animal tissues. In some cases, tumor cell lines are used, e.g., for estimating the toxic effects of NPs used in cancer chemotherapy. The type of cells is selected according to the potential route by which NPs enter the body. This may be oral uptake (mainly by ingestion), transdermal uptake (through the skin surface), inhalation uptake of NPs contained in the breathing air, or intentional NP injection in clinic. Intestinal epithelium cells (Caco-2, HT29, and SW480) are often used in experimental models for studying the toxicity of ingested NPs (Table 1). In these models, the kinetics of NP uptake by cells and the viability of cells upon the NP uptake are studied.

**Table 1:** Results of estimation of nanoparticle toxicity in experimental models of their oral uptake

Type of nanoparticles	Sizes	Concentration; incubation time	Cell line	Method of detection	Effects; conclusions	Reference
Ag, TiO2, and ZnO NPs	Ag, 20–30 nm TiO2, 21 nm ZnO, 20 nm	0.1, 1, 10, and 100 mg/ml; 24 and 48 h	Caco-2 SW480	МТТ assay; ELISA; LDH assay; ROS assay	Cell death (ZnO NPs are more toxic). ROS production. Release of IL-8 (Caco-2 cells produce more IL-8 than SW480 cells).	[136]
Latex NPs and microbeads	50 nm and 100 nm	10–1000 μg/ml; 4 h	Caco-2 Calu-3	MTS assay; LDH assay; transepithelial electrical resistance measurement; confocal microscopy	Cell death (positively charged NPs are more toxic). Release of LDH from cells. Penetration of the NPs into cells. Transport of the NPs through the epithelium layer (16–24% of the microbeads and < 5% of the NPs entering a cell monolayer are transported through it).	[137]
Spherical (SNPs) and rod-shaped (RNPs) CuO NPs	SNPs: diameter, 40 ± 16 nm RNPs: thickness, 10 ± 3 nm; length, 74 ± 17 nm	5–100 mg/ml; 24, 48, and 120 h	Caco-2 A549 SZ95 N-hTERT	MTS assay; PCR; immunoblotting; ELISA	Decreased cell viability (RNPs are more toxic). Expression of genes encoding proinflammatory cytokines. The transcript profile varies depending on the type of NPs: CD3E in the case of RNPs; IL-1a, IL-9, and CD86 in the case of SNPs.	[138]
CdTe QDs	3.5–4.5 nm	1, 0.1, and 0.01 mg/l; 24 h	Caco-2	Fluorescent microscopy; transepithelial electrical resistance measurement	Cell death related to penetration of QDs into them. Decreased TEER at a QD concentration of 0.1 mg/l.	[139]
MgO, ZnO, SiO2, TiO2, and carbon black NPs	MgO, 8 nm ZnO, 10–20 nm SiO2, 14 nm TiO2, ˂10–300 nm Carbon black, 14 nm	20 and 80 mg/cm2; 24 h	Caco-2	WST-1; LDH assay; DNA comet assay; glutathione level measurement	Decreased cell viability. Release of LDH from cells. Double-strand DNA breaks and oxidative damage of DNA. Decreased glutathione level.	[140]
Ag nanorods	Length-to-diameter ratio, 4:1	0.4 nM; 4 days	HT29	МТТ assay; cell count	Cytotoxicity is related to surfactants on the nanorod surface.	[141]
CdSe QDs	1.4–2.5 nm	2–200 pM; 24 h	Caco-2	МТТ assay; test for cell culture adhesion	Cytotoxicity is observed at a concentration of 200 pM because of the release of Cd from QD cores.	[142]
Multiwalled carbon nanotubes modified with COOH groups	1.4 ± 0.1 nm	5–1000 μg/ml; 24 h	Caco-2	MTS assay; LDH assay; staining with neutral assay; staining with trypan blue	Cell death at a nanotube concentration higher than 100 μg/ml.	[143]
Polystyrene NPs modified with COOH and NH2 groups	20–40 nm	0.3–12 nm;16 h	Caco-2	Transepithelial electrical resistance measurement confocal microscopy; caspase 3 assay; fluorescent microscopy	The NPs modified with COOH are more readily absorbed by cells. Decreased cell viability (the negatively charged COOH-modified NPs are more toxic).	[144]
VO nanotubes	Diameter, 15–100 nm	0.1–0.5 mg/ml; 4–24 h	Caco-2	Neutral red assay	Cell death caused by the nanotubes.	[145]
Polystyrene NPs modified and not modified with carboxylic acids	20 and 40 nm	0.3–6.6 nM; 4–16 h	Caco-2	L/D cell assay; clustering analysis; apoptosis assay	Decreased cell viability. Carboxylic acid-functionalized NPs decrease the cell viability more quickly and strongly.	[144]

The NPs that serve as carriers of drugs or contrast agents, or those used for imaging, are administered by injection. The toxicity of these NPs is studied in primary blood cell cultures. Most commonly, hemolysis, platelet activation, and platelet aggregation are estimated. In addition to primary blood cell cultures, cultured HUVECs, mesenchymal stem cells, mononuclear blood cells, and various tumor cell lines (HeLa, MCF-7, PC3, C4-2, and SKBR-3) are used (Table 2).

**Table 2:** Results of estimation of nanoparticle toxicity in experimental models of their intravenous administration and the consequences of interaction of nanoparticles with cells of various organs

Type of nanoparticles	Sizes	Concentration; incubation time	Cell line	Method of detection	Effects; conclusions	Reference
FeО NPs modified and not modified with polyethylene oxide triblock copolymer (PEO-COOH-PEO)	10 nm	1–5 mg/ml; 48 h	PC3 C4-2 HUVECs	MTT assay; confocal microscopy	Decreased viability of all cell types. NP uptake by cells. The surface-modified NPs are more toxic than NPs without shells.	[146]
SiO NPs modified and not modified with COOH, NH_2_, and OH	30 and 70 nm	1–6000 μg/ml; 24 h	HUVECs	MTS assay; ELISA; LDH assay; fluorescent microscopy	The unmodified NPs do not affect cell viability substantially. The modified NPs cause death of an insignificant proportion of cells. The cell state (static or dynamic) does not affect cell viability upon interaction with the NPs but affects internalization of the NPs (cells in the dynamic state absorb the NPs more readily).	[147]
CuS nanoplates	Length, 59.4 nm; thickness, 23.8 nm	1–400 μg/ml; 24 and 48 h	HUVECs RAW 264.7 KB HeLa	WST-8; confocal microscopy; scanning electron microscopy (SEM)	HUVEC viability is considerably more decreased in the presence of the NPs at concentrations higher than 100 μg/ml compared to KB and HeLa cells. The NPs penetrate only into RAW 264.7 cells. The NPs do not cause significant changes in the cytoskeleton of cells of any line.	[148]
Se NPs modified and not modified with Ru(II) polypyridyl	100 nm	1–50 μg/ml; 12 and 24 h	HUVECs HepG2 SW480 PC3 MCF-7	Immunoblotting; confocal microscopy; MTT assay; flow cytometry	The modified NPs are 20 to 6 times more toxic for all cell lines than the unmodified NPs. The modified NPs inhibit the proliferation and migration of HUVECs and formation of microtubules in them. The modified NPs are effectively absorbed by HUVECs and HepG2 cells.	[149]
Ag NPs	35, < 100, and 2000–3500 nm	22, 70, 220, 700, and 2200 μg/ml; 3.5 h	Human red blood cells	Hemolytic test	The NPs lyse a larger proportion of red blood cells compared to micrometer-sized particles. Hemolysis is enhanced at NP concentrations of 220 μg/ml and higher.	[150]
Hydroxyapatite NPs modified and not modified with indocyanine green and Gd^3+^	50 nm	50–250 mg/ml; 48 h	Mononuclear blood cells Mesenchymal stem cells	MTT assay; hemolytic test; test for platelet activation and aggregation; flow cytometry	The NPs are nontoxic for both stem cells and mononuclear cells of peripheral blood, do not cause platelet aggregation or activation, and do not induce inflammatory or immune response.	[151]
SiO NPs	100 nm	1–100 μg/ml; 24 and 48 h	HeLa 3T3	MTT assay; trypan blue test; flow cytometry; LDH assay; SEM; ROS assay	The NPs are low-toxic, decreasing the cell survival by more than 20% only at a concentration of 100 μg/ml. The NPs do not cause apoptosis, ROS generation, or serious morphological changes in cells at concentrations lower than 100 μg/ml.	[152]
CdTe QDs modified with mercaptosuccinic acid	4 nm	0.1–100 μg/ml; 24 h	HUVECs	MTT assay; flow cytometry; ROS assay	The QDs are toxic for HUVECs. The QDs increase the intracellular ROS level and activate apoptosis.	[153]
CdTe/CdSe/ZnSe QDs modified with mercaptoundecanoic acid	19.8 ± 5 nm	1.25–60 μg/ml; 1 and 24 h	HepG2, SKBR-3 MCF-7	Alamar blue assay; fluorescent microscopy; confocal microscopy	The QDs are nontoxic for all cell lines except HepG2 (for HepG2 cells, they are toxic at a concentration of 15 μg/ml). Morphological changes are also observed only in HepG2 cells.	[154]

The toxicity of inhaled NPs is studied using the cell lines modeling different tissues of the respiratory system, e.g., A549 and C10 cells of pulmonary origin, alveolar macrophages (RAW 264.7), various epithelial cells and fibroblasts (BEAS-2B, NHBE, 16-HBE, SAEC), as well as human monocytes (THP-1) (Table 3).

**Table 3:** Results of estimation of nanoparticle toxicity in experimental models of their inhalation uptake

Type of nanoparticles	Sizes	Concentration; incubation time	Cell line	Method of detection	Effects; conclusions	Reference
ZnO NPs	288.2 ± 2.4 and 265.7 ± 3.6 nm	4, 10, 25, 50, 100, 250, 500, and 1000 μg/ml; 6 and 24 h	С10	МТS assay; fluorescent microscopy; ROS assay	Decrease in cell viability after 6 and 24 h of incubation. Oxidative stress because of leakage of Zn ions.	[155]
Cu, CuO, ZnO, TiO_2_, Ti, Ag, Co, Ni, NiO, ZrO_2_, ZrO_2_+Y_2_O_3_, steel, Al_2_О_3_, SnO, WC, and CeO_2_ NPs	< 500 nm	1–10,000 μg/ml; 24 h	A549 THP-1	MTT assay; neutral red assay	The Cu and Zn NPs are the most toxic. The Al, Ti, Ce, and Zr NPs are low-toxic. The WC NPs are nontoxic. Toxicity in the NPs is not related to their shape, diameter, or surface area.	[156]
CuO NPs	50 nm	1–40 μg/ml; 24 h	A549 SAEC	WST-8; SEM; flow cytometry; confocal microscopy; immunoblotting; DNA microarray analysis; real-time PCR	The NPs are highly toxic for both cell lines. The NPs strongly affect the cell cycle, inhibiting the genes responsible for proliferation. The NPs cause apoptosis of A549 and SAEC cells.	[157]
Carbon nanotubes	14, 25.7 ± 1.6, 14.84 ± 0.05, 10.40 ± 0.32, 84.89 ± 1.9, and 165.02 ± 4.68 nm	5–50 μg/cm^2^; 24 h	THP-1 Met5a	ELISA; trypan blue тест; ROS assay; flow cytometry	Decreased cell viability and induction of ROS production. Intense release of acute phase inflammatory cytokines (IL-1β, TNFα, and IL-6) and chemokines (IL-8) from THP-1 cells.	[158]
CdSe QDs modified with mercaptoundecanoic acid (MUA), mercaptopropionic acid (MPA), aminoundecanoic acid (AUA), or cysteamine (CA)	3, 5, and 10 nm	0.5, 5, 20, 80, and 160 μg/ml; 22 h	NHBE	WST-1; LDH assay; ELISA; fluorescent microscopy	The positively charged (AUA- and CA-modified) QDs are more toxic than the negatively charged (MUA- and MPA-modified) QDs. The negatively charged QDs enhance the expression of proinflammatory cytokine genes; the positively charged QDs induce changes in the genes involved in mitochondrion functions.	[159]
SiO_2_ and Fe_3_O_4_ NPs modified and not modified with sodium oleate; TiO_2_ and PLGA NPs modified with polyethylene oxide (PLGA-PEO)	PLGA-PEO, 140 nm; SiO_2_, 25 and 50 nm; TiO_2_, 21 nm; Fe_3_O_4_, 8 nm	0.6–75 μg/cm^2^; 24 and 48 h	16-HBE A549	WST-1; flow cytometry; real-time PCR	The PLGA and TiO_2_ NPs have no considerable effect on 16-HBE or A549 cell viability. The modified Fe_3_O_4_ NPs are more toxic than unmodified ones. The PLGA NPs induce ROS generation without affecting cell metabolism, viability, or cytokine production rate.	[160]
CdSe/ZnS QDs modified with COOH or NH_2_ groups (COOH-QDs and NH_2_-QDs, respectively)	4–10 nm	2.5, 5, 7.5, 10, 15, and 20 nM; 1–3 cell cycles	BEAS-2B HFF-1 TK6	Flow cytometry; transmission electron microscopy (TEM); ELISA; ROS assay; calculation of cell population doubling time; fluorescent microscopy	The rate of QD uptake is considerably higher in BEAS-2B and TK6 cells. The COOH-QDs are more readily absorbed by cells. TK6 and HFF-1 cells are more sensitive to the QDs (a high toxicity is observed at concentrations higher than 15 nM) than BEAS-2B cells (a high toxicity is observed at concentrations higher than 20 nM). Minor changes in the ROS level are observed only in HFF-1 cells in the presence of the COOH-QDs and in TK6 cells in the presence of the NH_2_-QDs.	[161]
InP/ZnS and CdSe/ZnS QDs	InP/ZnS, 11.3 ± 0.6 nm; CdSe/ZnS, 13.4 ± 0.7 nm	1, 10, and 100 pM and 1 and 5 nM; 24 and 48 h	A549 SHSY5Y	WST-8; LDH assay; glutathione level measurement; analysis of mRNA expression level; TUNEL test	The CdSe/ZnS QDs damage the cell membrane, enhance the expression of detoxification enzyme genes, increase the antioxidant level, cause DNA damage, and disturb Ca^2+^ homeostasis in cells. The InP/ZnS QDs are less toxic.	[162]
CeO_2_ NPs	15, 25, 30, and 45 nm	5, 10, 20, and 40 g/ml	BEAS-2B	MTT assay; glutathione level measurement; MTT assay; ROS assay; caspase 3 assay; fluorescent microscopy	Cell death mediated by ROS generation. The NPs are absorbed by cells and localized in the perinuclear space.	[55]

The toxicity of NPs that enter the body transdermally is usually studied in keratinocytes, fibroblasts, and, more rarely, sebocytes (cells of sebaceous glands) (Table 4).

**Table 4:** Results of estimation of nanoparticle toxicity in experimental models of their transdermal uptake

Type of nanoparticles	Sizes	Concentration; incubation time	Cell line	Method of detection	Effects; conclusions	Reference
Ag NPs modified with digallic acid (DA-Ag) and not modified	DA–Ag, 13, 33, and 46 nm;Ag, 10–65 nm	1–10 μg/ml; 24 h	291.03C RAW 264.7	Neutral red assay; flow cytometry; TEM; [^3^H]thymidine staining of DNA; estimation of mitochondrion activity (JC-1 test)	The Ag NPs decrease the proliferation rate of both cell lines. The NPs enhance ROS generation in RAW 264.7 cells. RAW 264.7 cells absorb the 10- to 65-nm Ag and 33 and 46-nm, DA-Ag NPs, whereas 291.03C cells absorb only the 13-nm DA–Ag NPs. The Ag NPs suppress the production of TNFα by RAW 264.7 cells and enhance its production by 291.03C cells. The 33- and 46-nm DA-Ag NPs are the least toxic.	[163]
Si NPs modified with Al_2_O_3_ (Al_2_O_3_-Si) and Na (Na-Si)	Al_2_O_3_-Si, 21 nm; Na-Si, 30 nm	40–800 μg/ml; 72 h; 7 days	3T3-L1 WI-38	WST-1; LDH assay; glutathione level measurement	The Al_2_O_3_-Si NPs are nontoxic for 3T3-L1 cells and slightly toxic for WI-38 cells (a small decrease in viability at an NP concentration of 250 μg/ml). The Na-Si NPs are toxic for both 3 T3-L1 and WI-38 cells.	[164]
ZnO NPs modified with NH_2_ groups	20 nm	1–50 μg/ml; 0.5–24 h	HaCaT SCCE02	MTT assay; immunoblotting; ELISA; TEM; real-time PCR; ROS assay; fluorescent microscopy	Decreased viability of both cell lines at NP concentrations of 10 μg/ml and higher. Induction of oxidative stress through activation of MAP kinase signal pathways (ERK, JNК, and p38). Enhanced expression of Egr-1 and, as a consequence, TNFα.	[165]
Multiwalled carbon nanotubes (MWCNTs)	Diameter, 12 nm	100 μg/ml	SZ95 IHK	MTS assay; LDH assay; transepithelial electrical resistance measurement; [^3^H]thymidine staining of DNA; TEM	MWCNTs are toxic only for IHK cells. The TEER is unchanged, which indicates that MWCNTs do not affect the tight junctions of epidermal cells.	[166]
ZnO and TiO_2_ NPs	268.1 ± 11.2 and 414.9 ± 4.5 nm	0.5–10 μg/ml; 24, 48, and 72 h; 3 months	NCTC2544	MTS assay; scanning electron microscopy; ROS assay; flow cytometry	Decrease in viability upon incubation in the presence of the ZnO NPs at concentrations higher than 15 μg/ml for 24–72 h. Prolonged incubation causes changes in cell morphology and affects the cell cycle. The TiO_2_ NPs are nontoxic. The NP toxicity is related to the release of metal ions inducing oxidative stress.	[167]
CdSe/CdS NPs modified with polyethylene glycol	39–40 nm	0.3125–10 nM; 24 and 48 h	NHEK	Confocal microscopy; TEM; flow cytometry; atomic emission spectroscopy	Decreased viability at NP concentrations higher than 1.25 nM. Enhanced IL-8 and IL-6 production.	[168]
NaYF_4_ NPs modified with different compounds	94–550 nm	62.5 and 125 μg/ml; 24 h	HaCaT Human skin fibroblasts	MTT assay; confocal microscopy; fluorescent microscopy	The NPs coated with polyethyleneimine (PEI), poly(lactide-co glycolide) (PLG), and PLG + dextran sulfate are the most toxic (52, 61, and 72% viable cells, respectively). The NPs are nontoxic for fibroblasts. Hydrophilic NPs are the least toxic and are the most readily absorbed by the cells.	[169]
TiO_2_ NPs	124.9 nm	0.008–80 μg/ml; 6, 24, and 48 h	A431	MTT assay; Bradford protein assay; flow cytometry; glutathione level measurement; lipid peroxidase assay; DNA comet assay; ROS assay	A slight decrease in cell viability after 48 h of treatment. DNA damage with ROSs and micronucleus formation.	[170]
Polyamidoamine (PAMAM) dendrimers	4.5, 5.4, and 6.7 nm	0.01–21 μM; 24 h; 8 days	HaCaT SW480	MTT, clonogenic, Alamar Blue, and neutral red assays	The toxicity of the dendrimers linearly increases with increasing both their zeta potential and their size.	[171]

#### Co-cultured Cell Lines and 3D Cell Cultures

Although the majority of in vitro nanotoxicity studies are carried out on cell monocultures, studies using two other approaches are increasingly often reported in the literature. One of them is co-culturing of several types of cells; the other is the use of 3D cultures. The rationale for these approaches is the need for more realistic models of mammalian tissues and organs. For example, co-cultured Caco-2 epithelial colorectal adenocarcinoma cells and Raji cells (a lymphoblast cell line) have served as a model of the human intestinal epithelium in experiments on the toxicity of silver NPs [104]. A co-culture of three cell lines derived from lung epithelial cells, human blood macrophages, and dendritic cells has been used as an experimental model in a study on the toxic effects of inhaled NPs [105]. A model of skin consisting of co-cultured fibroblasts and keratinocytes has been suggested [106].

It is known that the cell phenotype, as well as cell functions and metabolic processes, is largely determined by the complex system of cell interactions with other cells and the surrounding extracellular matrix [107]. Therefore, many important characteristics of cells with an adhesive type of growth in a monolayer culture substantially differ from those of the same cells in the living tissue; hence, conclusions from many experiments on the NP toxic effects on cells growing in a monolayer are somewhat incorrect [108]. Experimental 3D models of tissues and organs have been used for analysis of NP toxicity and penetration into cells in several published studies. For example, there are 3D models based on polymer hydrogels [109] and models constructed in special perfusion chambers containing a semipermeable membrane to which the cells are attached. Li et al. and Lee et al. [110, 111] used multicellular spheroids about 100 μm in size to obtain a 3D model of the liver and compare the toxicities of CdTe and Au NPs in experiments on this model and a monolayer culture of liver cells [111]. The results obtained using the 3D model were more closely correlated with the data obtained in experiments on animals, which indicates a considerable potential of this approach for adequate and informative testing of NP toxicity.

#### In vivo Study of Nanoparticle Toxicity

In addition to the study of multilayered and 3D cell cultures, the behavior of NPs in the living body is being extensively studied. Since these studies are focused on the biomedical applications of NPs, the NP toxicity for living organisms remains an important issue. Although NPs are highly promising for various clinical applications, they are potentially hazardous. This hazard cannot be estimated correctly in vitro, following from the comparison of the in vivo and in vitro effects of NPs.

Titanium dioxide (TiO_2_) particles are among the most widely used NPs, in particular, in environment protection measures. Therefore, it was exceptionally important to estimate their toxicity in the case of a 100% bioavailability, namely, in experiments with their intravenous injection to experimental animals. This study has been performed by Fabian et al. [112]. Experimental animals (rats) were injected with a suspension of TiO_2_ NPs at a dose of 5 mg/kg, and their biodistribution, as well as the general condition of the animals, was monitored. The results have shown that the animals exhibit no signs of ailment or disorder, nor is inflammation or another manifestation of a toxic effect observed, within 28 days. This suggests that TiO_2_ NPs are relatively harmless.

Silver NPs are another example of NPs potentially useful in medicine, owing to their antimicrobial activity. Their toxicity and biodistribution were analyzed in an experiment where CD-1 mice were intravenously injected with 10 mg/kg of silver NPs of different sizes (10, 40, and 100 nm) coated with different shells. Although each type of NPs was found to cause toxic damage of tissues, larger particles were less toxic, probably, due to their lower penetration capacity [113]. Asare et al. [114] estimated the genotoxicity of silver and titanium NPs administered at a dose of 5 mg/kg. They have found that silver NPs cause DNA strand breaks and oxidation of purine bases in the tissues examined. Gold nanoparticles have a similar effect [115]. They have been shown to be toxic for mice, causing weight loss, decrease in the hematocrit, and reduction of the red blood cell count.

Targeted drug delivery is one of the most important applications of NPs. In this case, it is also paramount to know their toxic properties, because the positive effect of their use should prevail over the negative one. Kwon et al. [116] have developed antioxidant NPs from the polymeric prodrug of vanillin. Their study has shown that the NPs have no toxic effect on the body, specifically the liver, at doses lower than 2.5 mg/kg. Similar results have been obtained for gelatin NPs modified with polyethylene glycol, which are planned to be used for targeted delivery of ibuprofen sodium salt [117]. The NPs have proved to be nontoxic at the dose that is necessary for effective drug delivery (1 mg/kg), which has been confirmed by measuring the inflammatory cytokine levels in the animals studied, as well as histological analysis of their organs.

**Fig. 2:**
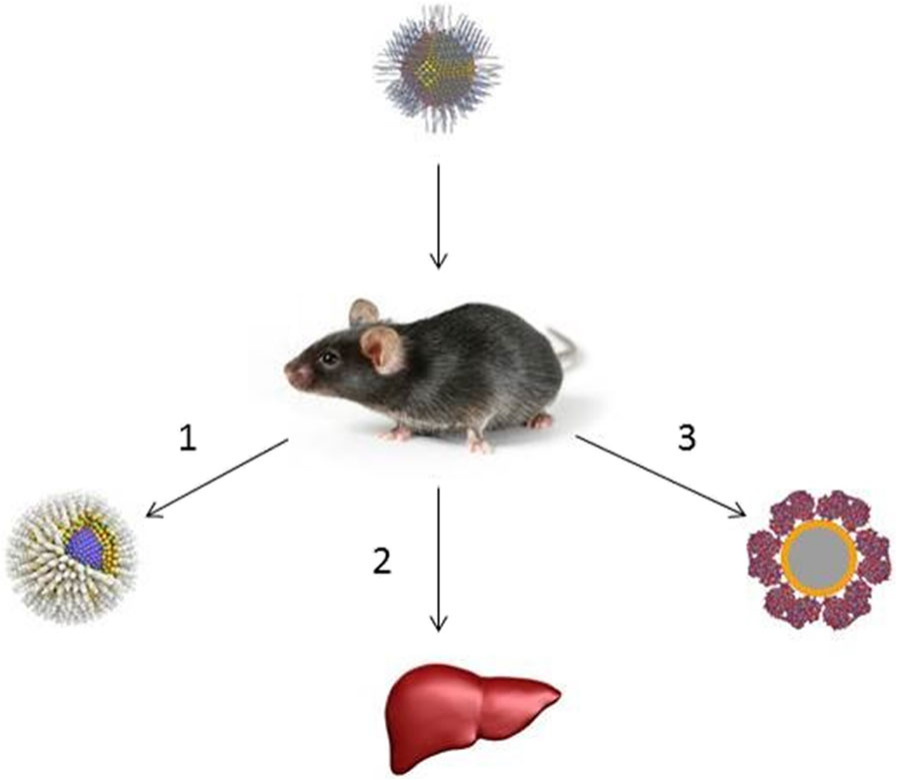
The possible reasons why quantum dots may be nontoxic in animal models. (1) The shell prevents the leakage of heavy metals into the body [129, 135]. (2) Quantum dots are localized in the liver and subsequently eliminated from the body [135, 173]. (3) The protein crown around quantum dots protects the body from heavy metals [132, 174]

Quantum dots are among the NPs that are most promising for medical applications (Fig. 2). However, they are potentially hazardous for human health, because they exhibit various toxic effects in both in vitro and in vivo experiments [118–122].

Toxic effects of QDs in vivo are usually studied in experiments on mice and rats [123]. A study on the toxicity of cadmium-based QDs for mice showed that QDs were distributed throughout the body as soon as 15 min after injection to the caudal vein, after which they accumulated in the liver, kidneys, spleen, red bone marrow, and lymph nodes. Two years after the injection, fluorescence was mainly retained in lymph nodes; in other organs, no QDs were detected [124]. It should be also noted that the fluorescence spectrum was shifted to the blue spectral region because of the destruction of the QD shell and changes in the shape, size, and surface charge of the QDs. This, however, occurred rather slowly, because the QDs were found to be nontoxic after their injection at the doses at which pure cadmium ions would have had a lethal effect. Similar results were obtained by Yang et al. [125]. Zhang et al. [95] showed that CdTe QDs predominantly accumulated in the liver, decreasing the amount of antioxidants in it and inducing oxidative stress in liver cells.

Cadmium and tellurium ions tend to accumulate in various organs and tissues upon degradation and decay of the cores of CdTe/ZnS QDs. Experiments on mice have shown that cadmium predominantly accumulates in the liver, kidneys, and spleen, whereas tellurium accumulates almost exclusively in the kidneys [126]. Ballou et al. [127] found that cadmium-containing QDs coated with polymer shells of polyacrylic acid or different derivatives of polyethylene glycol had no lethal effect on experimental mice and remained fluorescent for 4 months. СdSe/ZnS NPs also had no detectable pathological effect on mice [128]; however, the absence of distinct signs of pathology still does not mean that the QDs are absolutely nontoxic.

Hu et al. [129] found that lead-containing QDs had no toxic effect on mice for 4 weeks; however, this was most probably because the QDs studied were coated with a polyethylene glycol shell.

Since heavy metals contained in QDs are a factor of their toxicity, several research groups suggested that heavy-metal-free NPs be synthesized. For example, Pons et al. [130] synthesized CuInS2/ZnS QDs fluorescing in the near-infrared spectral region (at a wavelength of about 800 nm) and supposed that this composition would make the QDs nontoxic for experimental animals. Comparison of the effects of CuInS_2_/ZnS and CdTeSe/CdZnS QDs on regional lymph nodes in mice showed that the lymph nodes were only slightly, if at all, enlarged upon injection of the QDs not containing heavy metals, whereas injection of the CdTeSe/CdZnS QDs induced a distinct immune response in them [130]. QDs in which silicon was substituted for heavy metals also had no toxic effect on mice [131].

Even QDs containing heavy metals are often found to be nontoxic. One of the possible explanations is that QDs are coated with the protein crown upon entering the living body; this crown shields their surface and protects cells against damage [132]. Usually, the proteins that are included in the NP molecular corona are major serum proteins, such as albumin, immunoglobulin G (IgG), fibrinogen, and apolipoproteins [133]. Molecular corona also can influence on the interaction of NPs with cells. Zyuzin et al. have demonstrated that, in human endothelial cells, the NP protein corona decreases the NP nonspecific binding to the cell membrane, increases the residence time of NP in early endosomes, and reduces the amount of internalized NPs [134].

However, even in the absence of direct signs of intoxication in experimental animals, it remains unclear whether the use of QDs in medicine is safe for humans. In some cases, the QD toxicity was not detected in mice because the NPs were neutralized by the liver and accumulated in it [135]; in other cases, QDs coated with phospholipid micelles exhibited reduced toxicity owing to the shell [129]. Despite the extensive in vivo studies on QD toxicity, their use in biomedicine remains an open question. One of the main reasons is that all the delayed effects of QDs cannot be monitored in experimental animals, because their lifespan is as short as a few years, which is insufficient for complete elimination or degradation of NPs.

## Conclusions

The potential toxicity of NPs is the main problem of their use in medicine. Therefore, not only positive results of the use of NPs, but also the possible unpredictable negative consequences of their action on the human body, should be scrutinized. The toxicity of NPs is related to their distribution in the bloodstream and lymph stream and their capacities for penetrating into almost all cells, tissues, and organs and interacting with various macromolecules and altering their structure, thereby interfering with intracellular processes and the functioning of whole organs. The NP toxicity strongly depends on their physical and chemical properties, such as the shape, size, electric charge, and chemical compositions of the core and shell. Many types of NPs are not recognized by the protective systems of cells and the body, which decreases the rate of their degradation and may lead to considerable accumulation of NPs in organs and tissues, even to highly toxic and lethal concentrations. However, a number of approaches to designing NPs with a decreased toxicity compared to the traditional NPs are already available. Advanced methods for studying the NP toxicity make it possible to analyze different pathways and mechanisms of toxicity at the molecular level, as well as reliably predict the possible negative effect at the body level.

Thus, it is obvious that designing NPs that have small or no negative effects is impossible unless all qualitative and quantitative physical and chemical properties of NPs are systematically taken into consideration and a relevant experimental model for estimating their influence on biological systems is available.

## Abbreviations

FDA: Food and Drug Administration
IL-1β: Interleukin-1-beta
MRT: Magnetic resonance tomography
NP: Nanoparticle
QD: Quantum dot
ROS: Reactive oxygen species
SEM: Scanning electron microscopy
TEM: Transmission electron microscopy
TNFα: Tumor necrosis factor alpha
